# Effect of Educational Level on Oral Health in Peritoneal and Hemodialysis Patients

**DOI:** 10.1155/2009/159767

**Published:** 2009-03-10

**Authors:** Gulsen Bayraktar, Idil Kurtulus, Rumeyza Kazancioglu, Isil Bayramgurler, Serdar Cintan, Canan Bural, Mine Besler, Sinan Trablus, Halim Issever, Nilgun Aysuna, Oktay Ozkan, Alaattin Yildiz

**Affiliations:** ^1^Department of Removable Prosthodontics, Faculty of Dentistry, Istanbul University, 34093 Istanbul, Turkey; ^2^Department of Periodontology, Faculty of Dentistry, Istanbul University, 34093 Istanbul, Turkey; ^3^Department of Nephrology, Haseki Training and Research Hospital, 34087 Istanbul, Turkey; ^4^Department of Nephrology, Istanbul Training and Research Hospital, 34103 Istanbul, Turkey; ^5^Department of Public Health, Faculty of Medicine, Istanbul University, 34093 Istanbul, Turkey; ^6^Department of Nephrology, Faculty of Medicine, Istanbul University, 34093 Istanbul, Turkey; ^7^Department of Nephrology, Vakif Gureba Hospital, 34091 Istanbul, Turkey

## Abstract

*Background*. In previous studies, the oral and dental health statuses were compared in hemodialysis (HD) and peritoneal dialysis (PD) patients without taking into account the effect of educational levels on oral health. Hence we aimed to make a comparison of these parameters based upon the subjects educational levels. *Patients and Methods*. 76 PD (33 males, 43 females-mean age: 44 ± 12 years) and 100 HD (56 males, 44 females-mean age: 46 ± 14 years) patients were included. The number of decayed, missing and filled teeth were detected, DMFT index was calculated and plaque index (PI) values were assessed. *Results*. Significantly higher numbers of filled teeth (*P* < .001) and lower PI values (*P* < .01) in the PD group were detected with higher educational levels, whereas no significance was detected in the HD group. Higher DMFT index values were assessed in the lower educated and high school levels in PD than HD patients (*P* < .05). Higher numbers of filled teeth (*P* < .05) were detected in the secondary school level in PD patients. This difference was even more significant in the high school level (*P* < .001). *Conclusion*. We assume that PD patients, who were found to be in a higher educational level, are more caring for their oral health as compared to HD patients.

## 1. Introduction

Dialysis treatment aims to clear blood from toxins by using a semipermeable membrane in patients diagnosed with chronic renal failure (CRF). Either the patient own peritoneal membrane (peritoneal dialysis (PD)), or a semipermeable synthetic membrane (hemodialysis (HD)) is used for this purpose [1].

To date, there have been studies evaluating the oral hygiene and dental health status of mostly HD patients in comparison to healthy controls (Cs). The mostly observed oral findings are xerostomia and parotitis, which are believed to result from a combination of direct gland involvement, chemical inflammation, dehydration, and mouth breathing [2–4]. Epstein et al. [5] reported that salivary sodium and chloride concentrations were not lower in dialysis patients, but potassium tended to be higher in these patient groups. These authors also detected significantly higher phosphate values, but no decrease in salivary calcium levels indicating that in the salivary gland there was any compensatory decrease in calcium when phosphate was elevated. An accelerated rate of calculus formation has also been reported in these patients, which was attributed to elevated salivary urea levels [3, 5, 6]. But on the contrary, this was reported to contribute to remineralization of decayed dental enamel, which leads to lower caries levels in this patient group [7]. However, conflicting data exists on the prevalence of caries in chronic renal failure (CRF) patients. Levy [8] reported a lower prevalence of caries in these patient groups, but there are also studies which claim to detect higher [6, 9] decayed (D), missing (M), and filled (F) teeth index (DMFT) scores which were developed to determine the oral and dental health statuses [10], as well as studies reporting comparable results [11, 12]. Furthermore, several studies [13–16] evaluating plaque accumulation in CRF patients attributed higher plaque indices (PIs) in the oral neglect of these patients.

Detecting oral problems in dialysis patients is of utmost importance for preventing potential infections [4, 17] because recent studies of the healthy population have shown that oral infections are associated with an increased risk of systemic complications like atherosclerosis [18], chronic obstructive pulmonary disease [19], and adverse pregnancy outcomes [20]. Due to the usage of the patient own peritoneal membrane during PD therapy, they are demanded to complete a hygiene training program at the onset of therapy in order to prevent an infection of the membrane. In this training program, the patients are instructed in the rules of PD therapy and in maintaining general hygiene. In patients elected for the PD instruction program, educational level is usually not a selection criterion. In spite of this fact, people with higher educational level are obviously more capable of completing this program.

In our literature search, one study was found evaluating the effect of educational level on dialysis choice amongst other factors like age, gender, and clinical parameters [21]. But none of these studies aimed at determining the relationship between the educational level and dental parameters or oral hygiene status of these patients.

Hence, it was the aim of this study to analyze and compare the effect of educational level on the number of decayed, missing and filled teeth, and DMFT index and PI values in a group of PD and HD patients.

## 2. Subjects and Methods

In this cross-sectional study, 76 PD patients (PD group-test group: 33 males, 43 females-mean age: 44 ± 12 years) and 100 HD patients (HD group-test group: 56 males, 44 females-mean age: 46 ± 14 years), who were followed up in Istanbul School of Medicine and Faculty of Dentistry out-patient clinics in 2000–2005, were included. All participants gave informed consent to take part in this study.

Patients on dialysis for more than 6 months were included in the study. The duration of dialysis was 4 hours per session with 300–350 mL/min blood flow rate and with a dialysate flow of 500 mL/min. All HD patients were dialyzed with standard bicarbonate containing dialysate bath. All PD patients were dialyzed using conventional lactate-buffered glucose-based PD solutions. Fifty five of PD patients were on CAPD (4 to 5 times exchanges per day with 2000 mL) and remaining 21 patients were on APD. Twenty eight patients had diabetes mellitus, and it was the reason for ESRD in 23 patients.

In 2 of HD patients (2%) and 1 of PD patients (1%), hepatitis B surface antigen was positive. Anti- HCV was found positive in 19 (19%) of HD patients and 12 (16%) of PD patients. Vascular access was AVF in 90 (90%) of HD patients and the remaining 10 (10%) had AVG. There was not any evidence for clinical findings of a vascular access infection.

### 2.1. Clinical Examination and Indices

Prior to clinical examination, a detailed medical history including the information about age, gender, and education level was recorded for all the participants. Intraoral evaluation of all patients was performed according to WHO standard methods and criteria [22]. Decayed, missing, and filled teeth were detected, and DMFT index was calculated using a mirror and a probe in all participants.

The thickness of microbial dental plaque on the tooth surface near the marginal gingiva was assessed using Silness and Löe Plaque Index (PI). After the teeth were dried, the microbial dental plaque was scraped by the Williams periodontal probe and evaluated by unaided eye [23].

### 2.2. Statistical Analyses

Statistical analyses were performed using a software (SPSS for Windows Software Package, Version 11.5.0; SPSS Inc., Chicago, Ill, USA).

Student's *t*-test was used to calculate the means and to assess the differences between the ages and biochemical parameters of PD and HD groups. The difference between the distribution of the gender of the PD and HD patients was analyzed with Pearson's chi-square test. Differences between time on dialysis of PD and HD groups were analyzed using Mann-Whitney U test.

The median (min-max) values and statistical difference of the number of decayed, missing, and filled teeth and DMFT index and PI values were assessed using Kruskal-Wallis test.

The differences of the number of decayed, missing, and filled teeth, and DMFT index and PI values between PD and HD groups based upon their educational levels was assessed using the one way Anova analysis. The level of significance was set at *P* < .05.

## 3. Results

No statistically significant differences were found between the distribution of age and gender as well as the means of time on dialysis among PD and HD groups. BUN (*P* < .001) and blood phosphor (*P* < .01) levels were statistically significantly higher in HD than PD patients, while creatinine and blood calcium levels were comparable in both groups (Table 1).

Distribution of educational level in the PD and HD groups is given in Table 2. The PD group was found to be composed of 37% of patients in the uneducated and primary school levels, whereas 34% of patients were in the high school level. In the HD group, these proportions were 72% and 7%, respectively. The secondary school level in both the PD and HD groups was found to be comparable (23% and 21%, resp.).

Median (min-max) values and statistical comparisons according to the educational levels of the PD patients for decayed, missing, and filled teeth; DMFT index; PI values are shown in Table 3. With higher educational levels, significantly higher numbers of filled teeth (*P* < .001), but lower PI values (*P* < .01), were detected.

Median (min-max) values and statistical comparisons according to the educational levels of the HD patients for decayed, missing, and filled teeth; DMFT index; PI values are shown in Table 4. No statistical differences were found between the educational levels and dental parameters or oral hygiene status.

Mean (±SD) values and statistical comparisons according to the educational levels of HD and PD patients for decayed, missing, and filled teeth; DMFT index; PI values are shown in Table 5. In the uneducated and primary school levels, DMFT index values were statistically significantly higher in PD than HD patients (*P* < .05). In the secondary school level, higher numbers of filled teeth, but lower PI index values, were detected in the PD than HD group (*P* < .05). In the high school level statistically significantly higher DMFT index scores (*P* < .05) and remarkably higher numbers of filled teeth (*P* < .001) were detected in the PD than HD group.

## 4. Discussion

It was shown in this study, conducted based upon the educational level of the patients, that with higher educational level the number of filled teeth was higher and oral hygiene status was improved statistically in only PD patients (Tables 3 and 5). Although not statistically significant, the oral hygiene status tended to improve also in the HD group with higher educational level (Table 4). Comparisons of dental parameters and oral health status according to educational levels between PD and HD groups revealed clearly that in all educational levels the number of filled teeth, DMFT index, and PI values was better in PD than HD patients. In the high school level, these differences were even remarkable (Table 5).

There were 5 times more patients in the higher educational level in the PD than HD group. Due to the instruction in hygienic rules PD patients get at the beginning of therapy, the authors believe that these people are expectedly more caring for their oral hygiene. The higher numbers of filled teeth as well as the lower PI values in the PD group are supporting this opinion.

In the present study, the comparison of the dental parameters and PI values in the HD group based upon their educational levels revealed no statistical significant differences. In spite of this, it was found that PI values were the lowest in the patient group with higher educational level (Table 4). It has also to be taken into account that patients in the uneducated and primary school levels are 2 times more in the HD than PD group. As a result of this, the authors believe that this patient group is neglecting their dental health and oral hygiene. Several studies [13–16] also confirm that oral hygiene negligence would cause higher plaque accumulation in this patient group. They explain this fact in that HD patients would be more dependent on a dialysis center than PD patients. HD patients are usually bound to a dialysis machine for approximately 4 hours several times a week. Galili et al. [15] claimed that HD patients would be in a depressive state because of their life threatening disease and thus would rather be uncooperative during dental treatment and would further neglect their oral hygiene. PD patients in contrast are usually capable of continuing their treatment outside a dialysis center. Because of the fact that this patient group has a more free life, they are supposed to care more for their oral hygiene. In our literature search, we were unable to find any study comparing dental parameters and oral hygiene statuses according to the educational level of these patient groups. Hence, we are unable to compare our results with previous studies.

In conclusion, regular dental visits and remotivation in plaque control is of vital importance to ensure optimal oral health in dialysis patients in order to prevent rejection of the allograft after transplantation as a result of oral infection [24].

The authors suggest that further studies on this topic should be conducted as multicenter approaches when necessary to get data from large patient groups.

## Figures and Tables

**Table 1:** Distribution of age, gender, and time on dialysis and laboratory findings among PD and HD groups.

	PD group	HD group	*P*-value
*n*	76	100
Age (year ± SD)	44 ± 12	46 ± 14	NS
Gender (male/female)	33/43	56/44	NS
Time on dialysis (months)	25 ± 29	27 ± 21	NS
BUN (mg/dL)	55 (30–91)	142 (32–282)	*P* < .001
Creatinin (mg/dL)	8.95 (2.30–16.50)	9.05 (3.90–147)	NS
Kt/V	2.25 (0.30–4.05)	1.11 (0.41–2.54)
Ca (mg/dL)	9.18 ± 0.9	9.04 ± 0.7	NS
P (mg/dL)	5.06 ± 1.2	5.95 ± 1.6	*P* < .01

*n*: Number of patients in the groups; PD group: Group of patients receiving peritoneal dialysis treatment; HD group: Group of patients receiving hemodialysis treatment; BUN: Blood urea nitrogen; Kt/V: Marker of dialysis adequacy (*k* (clearance) × *t* (dialysis time)/V (patient's body water)); NS: Statistically not significant (*P* > 0.05).

**Table 2:** Distribution of educational level in the PD ve HD groups . (*χ* ^2^ test, *P* < .001).

Educational level	PD group (*n*; %)	HD group (*n*; %)	Total
Uneducated and primary school	28 (%37)	72 (%72)	100
Secondary school	22 (%29)	21 (%21)	43
High school	26 (%34)	7 (%7)	33
Total	76	100	176

*n*: Number of patients in the groups; PD group: Group of patients receiving peritoneal dialysis treatment; HD group: Group of patients receiving hemodialysis treatment.

**Table 3:** Median (min-max) values and statistical comparisons for decayed, missing, and filled teeth, DMFT index, and PI values in the PD group.

PD group	Uneducated and primary school median (min-max)	Secondary school median (min-max)	High school median (min-max)	*P*
Missing	11 (0–30)	7 (0–32)	9 (0–23)	NS
Decayed	2 (0–7)	1 (0–7)	0 (0–7)	NS
Filled	0 (0–22)	3 (0–15)	6 (0–24)	*P* < .001
DMFT index	14 (0–37)	13 (1–32)	16 (3–39)	NS
PI	2 (0.38–3)	0.95 (0.08–3)	1.23 (0.25–3)	*P* < .01

PD group: Group of patients receiving peritoneal dialysis treatment; DMFT index: Decayed, missing and filled teeth index; PI: Plaque index; NS: Statistically not significant (*P* > .05).

**Table 4:** Median (min-max) values and statistical comparisons for decayed, missing, and filled teeth, DMFT index, and PI in the HD group.

HD group	Uneducated and primary school median (min-max)	Secondary school median (min-max)	High school median (min-max)	*P*
Missing	7 (0–26)	7 (0–16)	5 (1–21)	NS
Decayed	1 (0–9)	1 (0–5)	1 (0–6)	NS
Filled	0 (0–7)	0 (0–10)	0 (0–4)	NS
DMFT index	10 (0–30)	10 (0–25)	9 (1–22)	NS
PI	1.93 (0–3)	2.17 (0.43–3)	0.59 (0–3)	NS

HD group: Group of patients receiving hemodialysis treatment; DMFT index: Decayed, missing and, filled teeth index; PI: Plaque index; NS: Statistically not significant (*P* > .05).

**Table 5:** Mean (±SD) values and statistical comparisons for missing, decayed, and filled teeth, DMFT index, and PI in the PD and HD groups based upon their educational levels.

		PD group mean (±SD)	HD group mean (±SD)	*P*
Uneducated and primary school	Missing	12.4 ± 9	9.72 ± 7.8	NS
	Decayed	2.32 ± 2.3	1.71 ± 2.1	NS
	Filled	2.07 ± 5.1	0.92 ± 1.8	NS
	DMFT index	16.79 ± 10.5	12.35 ± 8.2	*P* < .05
	PI	2.06 ± 0.9	1.87 ± 0.9	NS

Secondary school	Missing	8.14 ± 7.6	7.62 ± 4.5	NS
	Decayed	1.55 ± 2.3	1.38 ± 1.3	NS
	Filled	4.05 ± 4.1	1.76 ± 2.7	*P* < .05
	DMFT index	13.73 ± 8.1	10.76 ± 5.4	NS
	PI	1.29 ± 0.9	2.09 ± 0.9	*P* < .05

High school	Missing	8.85 ± 5.3	6.57 ± 6.9	NS
	Decayed	1.31 ± 1.9	1.71 ± 2.2	NS
	Filled	7.42 ± 6.8	0.86 ± 1.6	*P* < .001
	DMFT index	17.58 ± 8.9	9.14 ± 6.5	*P* < .05
	PI	1.42 ± 0.7	1.33 ± 1.5	NS

PD group: Group of patients receiving peritoneal dialysis treatment; HD group: Group of patients receiving hemodialysis treatment; DMFT index: Decayed, missing and filled teeth index; PI: Plaque index; NS: Statistically not significant (*P* > .05).
